# Functional compressive mechanics and tissue biocompatibility of an injectable SF/PU hydrogel for nucleus pulposus replacement

**DOI:** 10.1038/s41598-017-02497-3

**Published:** 2017-05-24

**Authors:** Jingen Hu, Yang Lu, Ling Cai, Kwabena Gyabaah Owusu-Ansah, Gewen Xu, Feilong Han, Junjie Bao, Xiangjin Lin, Yiping Huang

**Affiliations:** 1 Department of Orthopedics, the First Affiliated Hospital, School of Medicine, Zhejiang University, NO. 79 Qingchun Road, Hangzhou, Zhejiang China; 2 School of Chemistry and Chemical Engineering & the Key Laboratory of Environment-friendly Polymer Materials of Anhui Province, Anhui University, NO. 111 Jiu Long Road, Hefei, China; 3 Department of Hepatobilliary and Pancreatic Surgery, the First Affiliated Hospital, School of Medicine, Zhejiang University, NO. 79 Qingchun Road, Hangzhou, Zhejiang China

## Abstract

In spinal degenerative disease, an injectable liquid hydrogel can fill in defect entirely, lessen the danger of implant relocation and following loss of disc height, minimizing the operative trauma. Here, we propose an injectable *in-situ* chemically cross-linked hydrogel by a two-component reaction of liquid silk fibroin with liquid polyurethane at physiological temperature conditions. Confined compression tests and fatigue tests were reported to assess physical properties of the hydrogel. Impact of different diameter on the biomechanical behaviours was tested to evaluate the clinical potentiality of the hydrogel for replacing nucleus pulposus. Degradation behaviours in different solutions and animal experiments were also investigated to examine the tissue biocompatibility of the hydrogel. The hydrogel modulus was affected by the hydrogel geometrical (diameter) parameters. SF/PU composite hydrogel can survive a million cycles, unconstrained fatigue resistance. More importantly, *in vivo* biocompatibility using New Zealand white rabbits, showed good biocompatibility over a three-month period in culture. Particularly, they showed the significant clinical merit of providing stronger axial compressive stiffness on confined compression test. Based on the outcomes of the present research, the SF/PU composite hydrogel may provide significant advantages for use in future clinical application in replacing nucleus pulposus field.

## Introduction

Disc degenerative disease (DDD) can affect the older adults and brings about badly physical disability^1–3^. Presently, there is no perfect therapy for degenerated spinal discs. Disease signs may develop by age or other factors affecting disease progression,including genetic and environmental risk factors. Many patients with severe symptoms may option to surgical operations involving motion segment fusion and discectomy. However, solid fusion is linked to the accelerated deterioration of neighbouring discs and an unsure result^4–8^. Discectomy is also linked to decreasing of disc height^9^. Nucleus replacement strategies have more than theoretically began an original therapy technique for DDD^10^.

This strategy has a slighter invading operating therapy and conserves the left disc substances (i.e., natural annulus fibrosus and endplates). Further, disc height and mobility are preserved and adjacent motion segment degeneration, which is often an outcome of solid fusion, is actively prevented. Maybe the nearly satisfying nucleus implant is the PDN-Solo^11–16^ device, manufactured by RayMedica. This nucleus has a hydrogel core and a woven polyethylene shell.

One actual weakness of PDN in these studies is obvious. It may incompletely fill the space remained by the ejected nucleus to leave potential axial spaces simulating human nucleus pulposus function. Filling the space completely is the requirement for withstanding mechanical loading states experienced under physiological delivery within the disc^17–19^ as well as minimizing PDN dislocation. Furthermore, PDN is greatly tougher than human nucleus pulposus, which could finally lead to endplate malfunction with sinking and smash. Inherent with PDN is the demand for an immensely invasive anterior surgical approach with an overlong healing time.

Recently, some researchers have focused on injectable *in situ* forming hydrogel. Most candidates as good biocompatible and mechanical properties can be altered to meet clinical needs^20–22^. The injectable hydrogel to be used for nucleus pulposus replacement has also attracted considerable attention, which can fill the degraded region entirely, lessen the danger of implant relocation and following loss of disc height, minimizing the operative defect. We priorly reported^23^ initial studies on an injectable silk fibroin/polyurethane composite hydrogel,which can fill in the whole potential space, making the novel implant more bio mimetic than the PDN. Our prior work has only focused on its unconfined compressive and rheological behavior and cell proliferation assay *in vitro*.

The ideal nature as a nucleus replacement substance was reported previously^24^. Such a material should satisfy one of the criteria: the hydrogel should have ample mechanic behavior and fatigue resistance properties. For example, the hydrogel should withstand consecutive stress, and the maximal stress experienced under physiological conditions. The aim of the present research was to further analyses the compressive mechanic features and the biocompatibility and stability *in vivo* of an SF/PU hydrogel and estimates the mechanic features and feasibility of the substance as a promising substitute for the degraded disc nucleus.

## Materials and Methods

This research is approved by the ethics committee of our institutional ethics committee and all methods were performed in accordance with the relevant guidelines and regulations. Synthesis of silk fibroin polyurethane hydrogel composite of three main steps: preparation of polyurethane prepolymers with terminal isocyanate groups, silk fibroin solution preparation and silk fibroin polyurethane composite hydrogel synthesis, as described in our previous research^23^.

### Confined compression testing

The hydrogel samples were immersed in phosphate buffered saline (PBS) solution (pH = 7.4) at 25 °C and unconfined compression tests were performed at 1 and 14 days after immersion. The beginning diameter and height of cylindrical hydrogel were determined using a vernier calliper. Swollen cylindrical samples (n = 3; 14.10 ± 0.10 mm diameter and 15.00 ± 0.00 mm height of the hydrogel at equilibrium swelling) were compressed using a strain rate of 100% strain/min in a Zwick Z2.5 compression bench with a 2500-N load cell (Zwick/Roell Z2.5, Zwick GmbH & Co., Ulm, Germany). Subsequently, load and displacement data were recorded at 10 Hz with the testXpert || software. A tangent compressive modulus was measured for each sample at 15%, 20%, and 25% compressive strain.

A confined compressive modulus was measured by applying a confined compression testing configuration fitted to a Zwick Z2.5 compression system (Zwick/Roell Z2.5, Zwick GmbH & Co., Ulm, Germany). A cylindrical plunger was compressed upon the hydrogel samples using a strain rate of 100% strain/min. The hydrogel (n = 3; 14.10 ± 0.10 mm diameter and 15.00 ± 0.00 mm height, after equilibrium swelling) was confined by a high-density polypropylene ring. The polypropylene ring was considered a harder body, as its modulus (approximately 10.0 GPa) is several orders of magnitude higher than that of the hydrogel. Load and displacement data were recorded at 10 Hz via the testXpert ‖ software. All data were collected and confined compressive modulus was measured at 4% compressive strain.

### Impact of different diameter on the biomechanical behaviours

To correlate different diameter with hydrogel biomechanical behaviours, samples (n = 3 for the 14.10 mm group with cylindrical samples of 15.00 mm height and 14.10 mm diameter, n = 3 for the 16.10 mm group with cylindrical samples of 15.00 mm height and 16.10 mm diameter) were performed to mechanical testing by using the Zwick Z2.5 compression system, swollen cylindrical samples of the hydrogel were compressed using a strain rate of 100% strain/min. All load and displacement data were collected at 10 Hz via the testXpert ‖ software. Values of stress–strain and tangent compressive modulus for all hydrogel samples were measured at 15%, 20%, and 25% compressive strain. The two groups have the same height and different diameter.

### Degradation of performance in different solutions

Hydrogel specimens were synthesized using silk fibroin and polyurethane, as described above. Tests were performed on hydrogel samples (n = 3 for the PBS group with cylindrical samples of 17.44 ± 0.26 mm height and 17.06 mm ± 0.42 diameter, after equilibrium swelling; n = 5 for the physiological saline solution group with cylindrical samples of 15.88 ± 1.31 mm height and 16.04 ± 1.01 mm diameter, after equilibrium swelling) after 1 day and 14 days immersion in PBS solution (pH = 7.4) at 25 °C and physiological saline solution (pH = 7.4) at 25 °C. The height and diameter of samples changes with time were calculated at nodes 1 day and 14 days.

### Fatigue tests

Hydrogel samples (n = 3) were synthesized, according to the procedures described above. The samples were compressed to axial strain of 15% for 1 million fatigue cycles at a frequency of 5 Hz in PBS solution (pH = 7.4) at 25 °C by using a biodynamic 5110 testing system. Tests were carried out on hydrogel samples (n = 3) under the same condition. Surfaces with the circular grooves were designed to use for keeping the hydrogel samples stationary on the testing device during testing. The axial strain of 15% is close to the actual strain corresponding to loading on the complete intervertebral discs (IVD) in normal physiological conditions^25, 26^. The dimensions of the hydrogel samples were calculated with a digital caliper before and after fatigue cycling testing. Samples were immersed back in PBS solution at 25 °C for up to 14 days, with dimensions calculated after 14 days.

### Animal experiments

Dry hydrogel were sterilized using iodophor solution and swollen in sterile PBS solution before implantation. Four adult New Zealand white rabbits (2.5–3 kg) (male:2) were anaesthetized and operated under sterile conditions. Small dorsal incision (1.5 cm–2 cm per incision) was made on the back and swollen hydrogels was implanted to assess the tissue reaction to the hydrogel samples. Eight small dorsal incisions (1.5 cm–2 cm per incision) were made. Four swollen hydrogel (approximately 3 mm diameter and 1 cm length/hydrogel column) were implanted (one swollen hydrogel/incision) in the paravertebral muscles on right side of the spine about 2 cm from the mid-line, whereas four blank control groups (only 1.5 cm-2 cm incision) weren’t implanted in the paravertebral muscles on left side of the spine about 2 cm from the mid-line. The implantation positions were marked on the fascia using non-absorbable surgical suture.

## Results

### Confined compression tests

**Figure 1:**
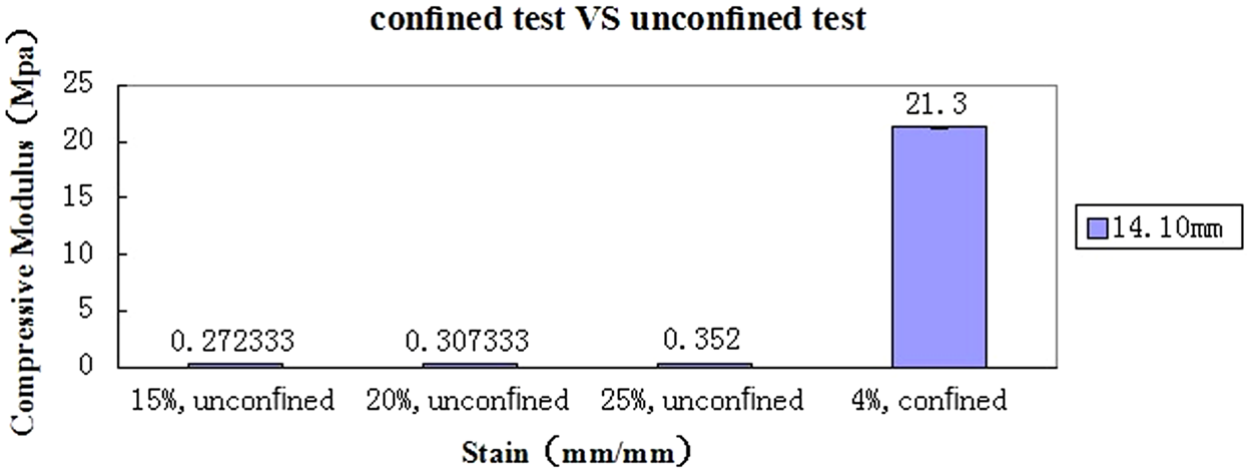
The tangent modulus values at 15%, 20% and 25% strain in the unconfined compression group was far lower than that of the confined compression group at 4% strain.

In all cases (Fig. 1), the unconfined compression group had lower values of the tangent modulus observed at 15%, 20% and 25% strain (3/3 of the confined compression group, 3/3 of the unconfined compression group) than the confined compression group at 4% strain. The tangent modulus values in confined compression group at 4% strain were 78.89-fold,71.00-fold, 60.86-fold higher than that of the unconfined compression group at 15%, 20% and 25% strain (21.30 ± 0.10 MPa vs.0.29 ± 0.00 MPa, p < 0.05; 21.30 ± 0.10 MPa vs.0.33 ± 0.00 MPa, p < 0.05; 21.30 ± 0.10 MPa vs.0.39 ± 0.00 MPa, p < 0.05, Fig. 1). The average confined modulus values were 21.30 MPa with polypropylene ring.

### Impact of different diameter on the biomechanical behaviours

**Figure 2:**
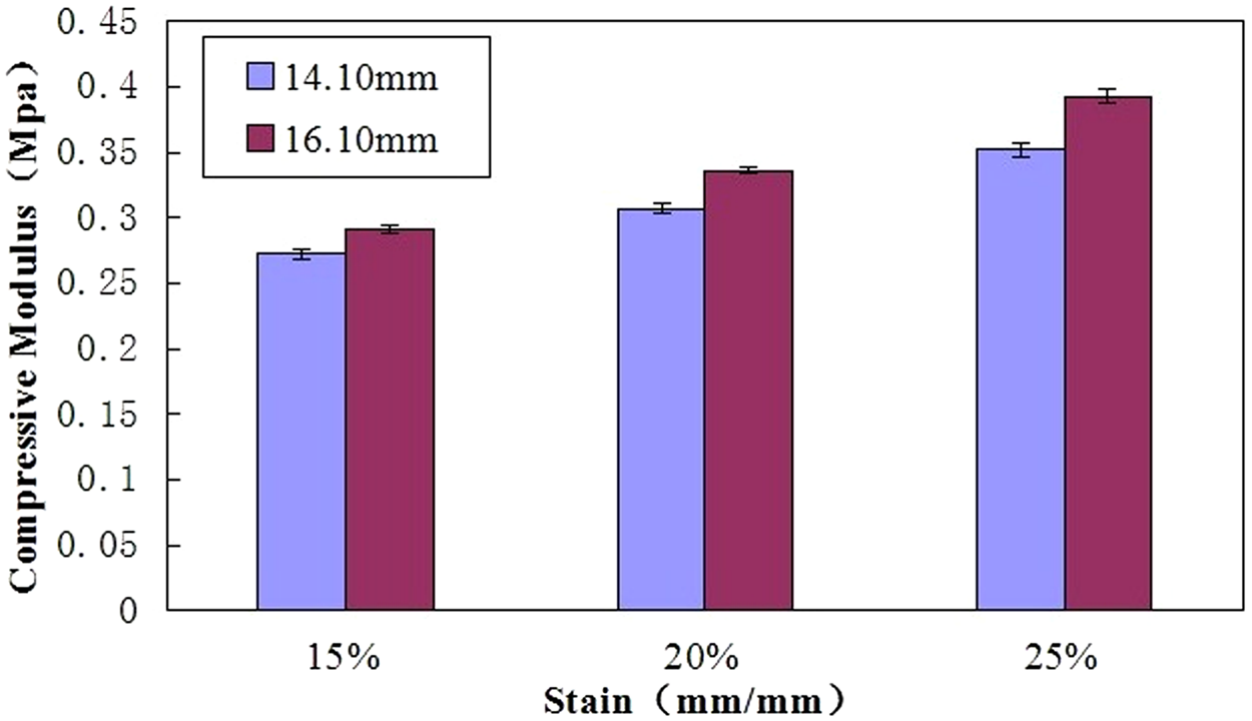
The tangent modulus values in 16.10 mm group at 15%, 20% and 25% strain were 0.07-fold, 0.10-fold, 0.11-fold higher than that of the 14.10 mm group. The unconfined compression modulus increases as the strain magnitude increases.

In all cases (Fig. 2), the 16.60 mm group had higher tangent modulus values at 15%, 20% and 25% strain (3/3 of the 16.10 mm group, 3/3 of the 14.10 mm group) than the 14.60 mm group, respectively. The tangent modulus values observed in 16.10 mm group at 15, 20 and 25% strain were 0.07-fold,0.10-fold,0.11-fold higher than that of the 14.10 mm group (0.27 ± 0.00 MPa vs.0.29 ± 0.00 MPa, p < 0.05; 0.30 ± 0.00 MPa vs.0.33 ± 0.00 MPa, p < 0.05; 0.35 ± 0.00 MPa vs.0.39 ± 0.00 MPa, p < 0.05, Fig. 2), respectively.

### Degradation of performance in different solutions

**Figure 3:**
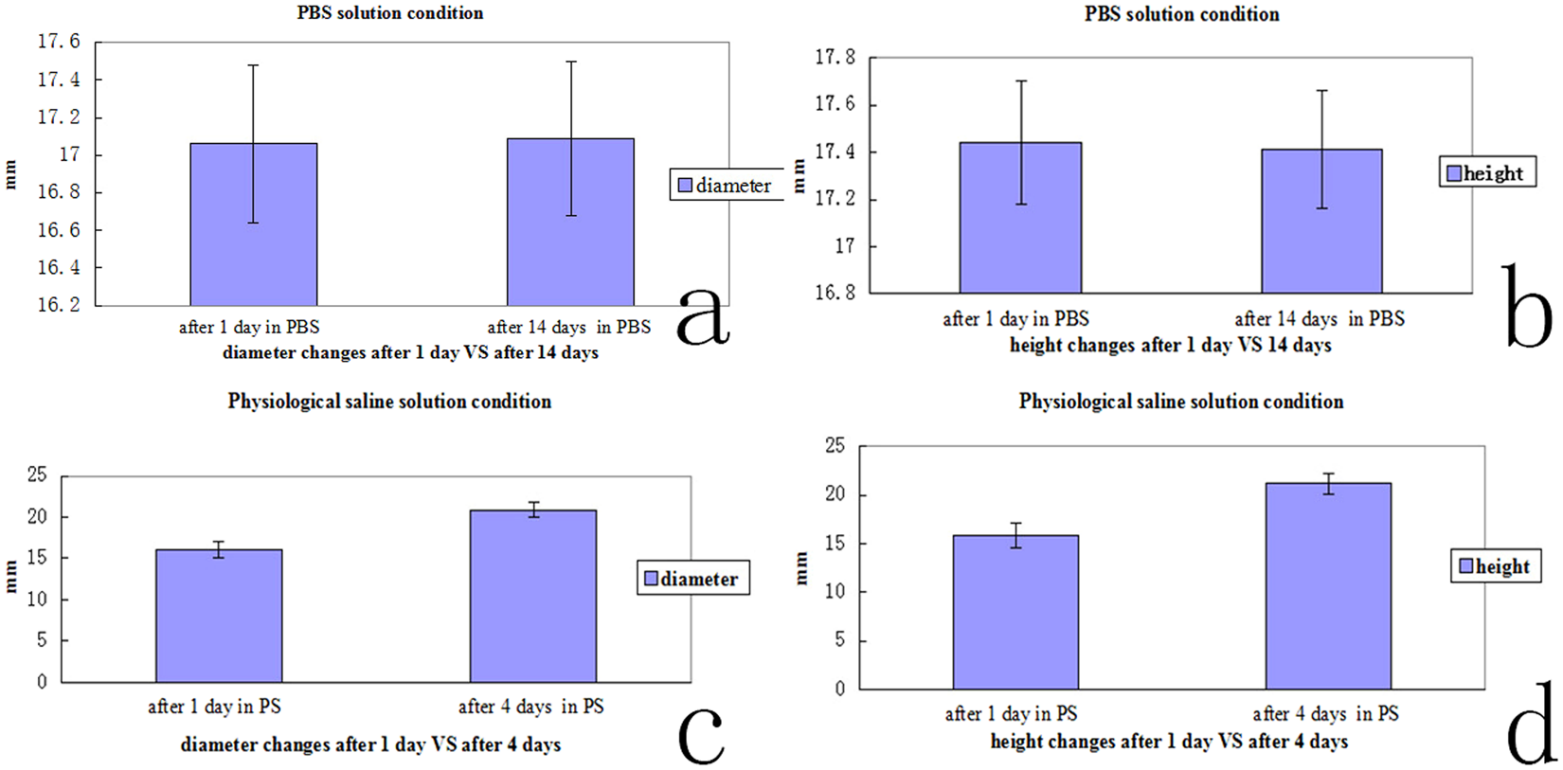
(**a**) The diameter for the PBS solution conditions (after 1 day immersion in PBS vs. after14 days immersion in PBS) was not significantly different (p > 0.05). (**b**) the height for the PBS solution conditions (after 1 day immersion in PBS vs. after14 days immersion in PBS) was not significantly different (p > 0.05). (**c**) the diameter of SF/PU hydrogel was significantly different (after 1 day immersion in the saline solution vs. after 4 days immersion in the saline solution, p < 0.05 at all the specimens levels). (**d**) the height of SF/PU hydrogel was significantly different (after 1 day immersion in the saline solution vs. after 4 days immersion in the saline solution, p < 0.05 at all the specimens levels).

In all the specimens, the height, diameter for the PBS solution conditions (Fig. 3a,b)(after 1 day immersion in PBS vs. after14 days immersion in PBS) was not significantly different (p > 0.05), indicating no material degradation of SF/PU hydrogel after 14 days immersion in PBS.

Immersion into the saline solution led to material degradation, compared to the PBS solution condition (Fig. 3c,d) and the height, diameter of SF/PU hydrogel was significantly different (after 1 day immersion in the saline solution vs. after 4 days immersion in the saline solution, p < 0.05 at all the specimens levels). SF/PU hydrogel were completely degraded when SF/PU hydrogel immersed in the saline solution after 14 days.

### Fatigue tests

Three hydrogel samples were tested under the fatigue tests conditions (compression-compression, 5 Hz, 106cycles). No fatigue failure was observed at all samples. The residual heights of samples, calculated at the end of the test, are collected in Table 1. The effect of the cumulative cyclic loading causes the hydrogel samples to creep.

**Table 1:** Sample Diameter and Height before and after fatigue cycling and after 14 days.

	Before fatigue cycling	After fatigue cycling	After 14 days.
	(n = 3)	(n = 3)	(n = 3)
Diameter(mm)	18.33 ± 0.27	19.73 ± 0.32^b^	18.31 ± 0.27^a^
Height(mm)	15.52 ± 3.08	13.97 ± 1.87^c^	15.42 ± 3.15^a^

^a^Statistical analysis showed that the height and diameter difference was not significant (p = 0.149,p = 0.073) before fatigue cycling and after 14 days.

^b^Statistical analysis showed that the diameter difference was significant (p = 0.006) before and after fatigue cycling.

^c^Statistical analysis showed that the height difference was not significant (p = 0.194) before and after fatigue cycling.

The loss of height was only small in the hydrogel samples, there was not statistically significant at the end of the experiment (p = 0.093). However, there was statistically significant in diameter changes (p = 0.006). There was not statistically significant in height (p = 0.161) and diameter (p = 0.073) changes after 14 days.

### *In vivo* biocompatibility

Macroscopic observations of all rabbits were recorded during the test. All rabbits survived until the end of test, and all test wounds healed by first intention. The wound healing process of the four adult New Zealand white rabbits seemed to be normal and the hydrogel sample implants couldn’t be seen on the back. No symptoms of infection or an aggravated inflammatory response were detected macroscopically in the implantation areas. Four rabbits were sacrificed after twelve weeks. After killing the rabbit, the hydrogels and some muscles around the implanted hydrogels were removed and fixed in buffered formalin. Serial cross embedded sections (3 um thick) of the specimens were cut and stained with haematoxylin-eosin (HE) for light microscopic examination. All the animals were processed according to the standard guidelines of Zhejiang University Ethics Committee (no. ZJU2007105002).

**Figure 4:**
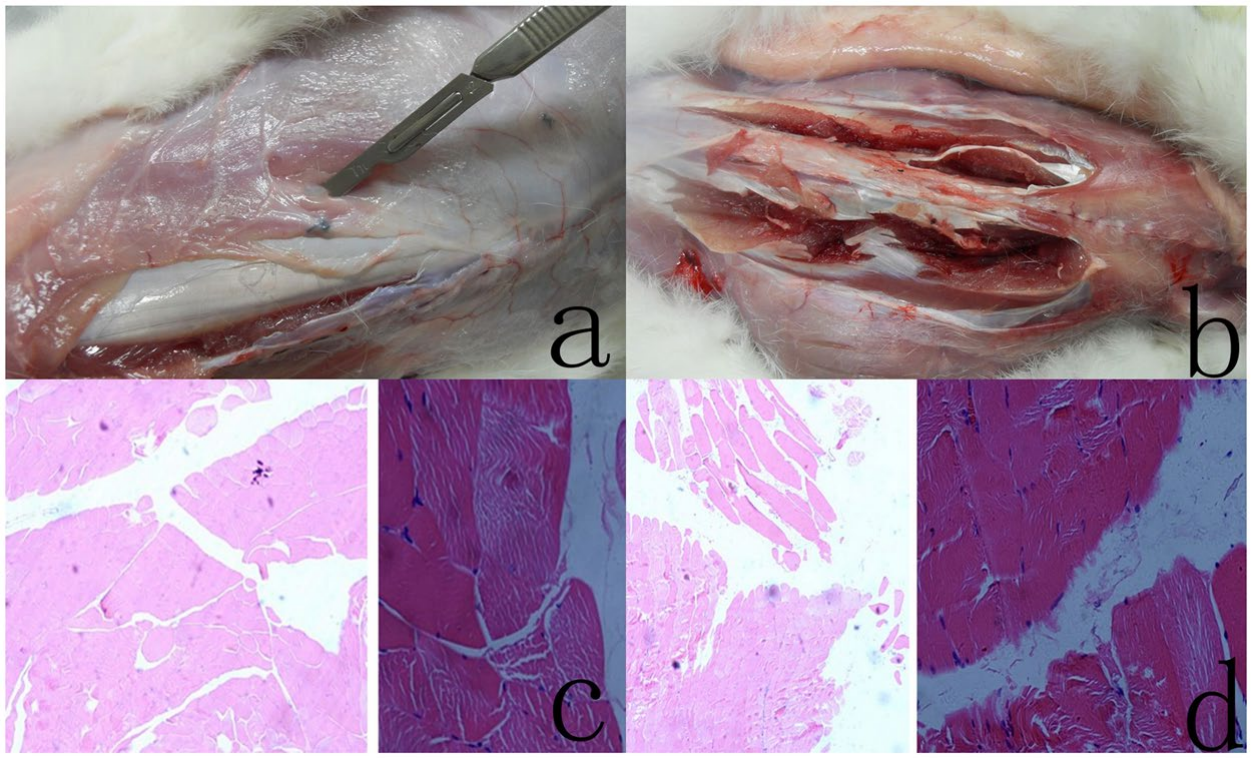
(**a**) The SF/PU hydrogel implanted for 3 months. The hydrogel is encapsulated in the muscle tissue of New Zealand white rabbits. After 3 months, the hydrogel is clear which can be seen at the center of the muscle tissue (beside the blade). The implanted SF/PU hydrogel maintained its shape at the implanted site for the full experimental observation period. the implants has not changed in visual appearance or dimensions after 3 months. (**b**) Animals were sacrificed at 12th week postoperatively; View of the muscle of the New Zealand white rabbit paravertebral muscles on left and right side of the spine. No inflammatory response was observed in the muscle. Neither infection signs nor pus formation was noticed grossly. (**c**) Microscopic appearance of the muscle of New Zealand white rabbit in the blank control groups. No inflammatory response was observed in the muscle. H & E stain, original magnification ×4 and ×20. (**d**) Microscopic appearance of the muscle of New Zealand white rabbits surrounding an implanted SF/PU hydrogel sample after 3 months. No minimal inflammatory response was observed in the surrounding muscle. H & E stain, original magnification ×4 and ×20.

Compared with the blank control groups (Fig. 4d), After 3 months, no minimal inflammatory response (Fig. 4e) was observed in the surrounding muscle. All implants isn/t surrounded by a thin fibrous capsule, containing linearly organized fibroblasts. Figure 4a,b showed representative macroscopic view of the hydrogels and rabbits after 3 months of muscle implantation. Similar morphology changes were observed for all hydrogel implants and rabbits. Basically, all hydrogel implants may triggered only minimal and comparable tissue response in 1 month after the implantation operation. A minimal inflammatory response may be observed in the surrounding muscle because of operation injury and steriled Iodophor or hydrogel. A minimal inflammatory response disappeared after 1 month. Again, no calcification was found around the sutures of implants. So both our swollen hydrogel implants performed with good biocompatibility as the blank control groups. Furthmore,after retrieval of the implants, the visual appearance or dimensions of the implants had not changed after 3 months. Evidently,for these SF/PU hydrogels to perform as permanent nucleus pulposus replacement, degradation is unacceptable.

The implanted SF/PU hydrogel maintained its shape at the implanted site for the full experimental observation period following removal of the hydrogel at 3 months (Fig. 4a). These results indicate that the SF/PU hydrogels maintained sufficient structural integrity to act as an artificial nucleus pulposus *in vivo*.

## Discussions

In functional requirement as a nucleus replacement, the implant would be in touch with the encircling annulus ring,perfectly in a whole contact condition^26, 27^ cylindrical unconfined hydrogel samples will further expand radially outwardly upon axial compression due to its high Poisson’s ratio. A cylindrical confined hydrogel samples in axial compression within a polypropylene ring has a relatively similar rigidity and will not directly transfer loads to surrounding neighboring ring, causing the ring structure to expand radially outwardly. The act of no increase in volume of this ring leads to a much higher compression modulus (a two-order-of-magnitude difference) observed compared with unconfined compression test.

Our elementary outcomes on the impact of diameter indicated that the values of tangent modulus increased with the increase of the hydrogel diameter. The 16.10 mm group may have more cross-linking (chemical and physical) than that of the 14.10 mm group owing to increase of the hydrogel diameter. The current research indicated the diameter (geometrical parameters) of hydrogel affected the hydrogel modulus. Our previous research^23^ also indicated that the height of hydrogel (geometrical parameters) affected the hydrogel modulus. There is striking evidence indicating that the implant geometrical (diameter and height) parameters affect the hydrogel modulus. In fact, the diameter of nucleus pulposus of intervertebral disc is larger than that of our sample (14.10 and 16.10 mm diameter). As a result, its tangent modulus values should be larger than that of the sample. This larger diameter of a normal nucleus compared to that of the sample and presumably creates larger tangent modulus in nucleus pulposus. The larger tangent modulus in the nucleus pulposus then allows the intervertebral disc to carry more loads, leading to higher stiffness of the intervertebral disc, via this synergetic interaction between annulus fibers and nucleus pulposus.

Previous studies using a finite element model^28^ predicted that the implant geometrical (diameter and height) parameters provide more to compressive mechanics of the disc compared to changes in implant modulus, for the examination ranges. They drew the conclusion that “compressive biomechanics were highly affected by implant volume (under-filling the nucleus cavity, line-to-line fit, or over-filling the nucleus cavity) with a greater restoration of compressive mechanics observed with the over-filled implant design” (Joshi 596). Arthur *et al*.^29^ provided the most convincing evidence to date that “fill of the nucleus cavity affects mechanical stability in compression, bending, and torsion of a spine segment, which has undergone nucleus replacement” (Arthur 687). These results showed that biomechanical function of implant depended upon the volume of material injected into residual nucleus cavity. Injectable hydrogel allows random surgical defects to be entirely filled. It allows personalized treatment according to the volume of every nucleus pulposus and loading in daily activities, since patients with different volume of every original nucleus pulpous have different spinal loading in daily activities. Injectable hydrogel can offer personalized treatment to recover original personalized biomechanical behavior of the anulus, whereas business, under-filling implants (such as PDN) could not do so. An injectable hydrogel (complete matching) was replaced in surgical defect cavity of lumbar disc with an integral annulus.

The electrolyte ingredient in PBS solution conditions is close to the human environment in the state. The SF/PU hydrogel did not show significant degradation in PBS, indicating that the SF/PU hydrogel may be stable in the human or animal body. The extent of degradation was noticeably related to the equilibrium degree of swelling of hydrophobically modified hydrogels^30^. They think by changing the degree of cross-linking, the length and content of the chains, it is possible to control the swelling and degradation behavior of the hydrogels.

Creep behaviour is often associated with materials under static load^31^. The cumulative fatigue affected creep behaviour in the materials because of polymeric chain relaxation, leading to the shape change. The materials could returned to their approximate original shapes after reversible creep strain, possibly due to a transient change in water content under cumulative fatigue test conditions. It is generally accepted that *in situ*, there is a synergistic effect between the nucleus pulposus and the annulus ring. This effect is almost impossible to bio mimetic accurately *in vitro*. We chose the unconstrained mechanical test, and the major purpose of the fatigue testing was to see if the hydrogels withstand a million fatigue cycles. One million fatigue cycles were chosen as a primary observe indicator. The constrained hydrogels are expected to demonstrate even better compared to those of currently unconstrained mechanical test. It should be noted that in human body, there is a 6–8 hour resting period every day, this gives our disc a chance to recover original shapes and functions after cumulative fatigue loading. This would also obtain improved performance if we give the hydrogels a desired attracting periodic resting time in the fatigue testing. The natural disc creeps, resulting in height loss under loading during the day because of body fluid flow^32^. This explains reasons why normal people cause decreased height of average 2 cm in the afternoon than in the morning. Overnight, the natural intervertebral discs recover original dimensions and the disc heights rewell^33^. To simulate this behavior, our study demands a hydrogel that could also returned to original dimensions after fatigue loading during the day.

The SF/PU hydrogel did not significant degradation in the muscle of New Zealand white rabbits, indicating that the SF/PU hydrogel should be stable in the human or animal body, Although the three-month submuscular implantation is too short to draw a very sound conclusion. Preclinical evaluation of new nucleus pulposus replacement is fundamental in proving hydrogel safety and efficacy. Cytotoxicity and biocompatibility evaluation should be carried out in all new hydrogel. Direct cell contact experiments are the most widely used ways of demonstrating toxicity. Direct cell contact experiments and standardized animal implantation studies are essential in biocompatibility evaluation prior to clinical researches^34^. We have previously demonstrated that the silk fibroin/polyurethane composite hydrogel causes no toxicity in direct cell contact experiment^23^. The present study confirms the good biocompatibility of silk fibroin/polyurethane composite hydrogel. Silk fibroin/polyurethane composite hydrogel material can thus be used safely as an artificial nucleus pulposus platform. Despite the good results of SF/PU hydrogel in practical artificial nucleus pulposus applications, few studies have evaluated the SF/PU hydrogel as an artificial nucleus pulposus. In this work, the SF/PU hydrogel was prepared through iodophor sterilization. The final results may have certain interference, because iodophor may increased the inflammation reaction.

In the current study, the rabbit model is to assess the biocompatibility. It is less convincing to use the rabbit model to study the physical-mechanical properties because of the difference in the biomechanical situation between human and rabbit. In our further study, we will study the *in vivo* implantation of the SF/PU hydrogel in baboon model with a minimally invasive surgical approach and longer follow-up.

### Concluding remarks

Injectable hydrogel treatment is a novel less invasive therapy for DDD, and the hydrogel plays an important role in minimally invasive treatment. However, designing an appropriate hydrogel is a difficult challenge. In the research, we propose a method for preparing the injectable *in situ* cross-linkable hydrogel, SF/PU, as a candidate material for nucleus pulposus replacement. Modulus of the hydrogel samples was affected by the hydrogel geometrical (diameter) parameters. Larger modulus was found for hydrogel samples undergone confined compression than for the hydrogel samples experimented in an unconfined condition. The results of confined compression researches demonstrated that the SF/PU composite hydrogel have adequate physical-mechanical capabilities that encourage further testing. Moreover, hydrogel has good stability in PBS. Additionally, animal experiments confirm the good biocompatibility of SF/PU composite hydrogel. The implanted hydrogels were able to maintain stability in animal body. In particular, no appreciable difference between in height and diameter changes of the hydrogel samples before and after 1 million cycles fatigue of compression-compression load to 15% strain was experimentally investigated. Fatigue tests showed the clinically important advantage of fatigue resistance in hydrogel, which are of absolute importance in recovering hydrogel after loading during daily life. These outcomes proposed that the injectable SF/PU composite hydrogel could be an appropriate material for nucleus pulposus implants in management of DDD.

There is a functional and load related difference between human that is vertical and rabbit that is horizontal spinal cord. Our future researches will contain animal experiment with horizontal spinal cord. Additionally, the pressive factor of injection and the hydrogel geometrical parameters will be studied to attain the desired complete matching of the hydrogel in the nucleus defect cavity. With our new approach, we may search for a better implant based on the improved physicochemical properties of the hydrogel.

In summary, these research outcomes provide evidence for the vast potential of utilizing SF/PU hydrogel for future research in this realm; boosting better application personalization of injectable SF/PU hydrogel as prosthetic appliances will help recover normal joint biomechanics. The new implant design for nucleus pulposus seems potential, with promising for animal trials and, ultimately, clinical application.

### Ethics approval

This study is approved by the ethics committee of the First Affiliated Hospital of Zhejiang University.
